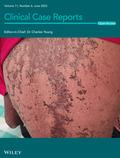# Cover Image

**DOI:** 10.1002/ccr3.7631

**Published:** 2023-06-27

**Authors:** Prajwal Pudasaini, Sushil Paudel, G. C. Sagar, Sadiksha Adhikari, Neeraj Thapa, Bibechan Thapa

## Abstract

The cover image is based on the Case Report *Extensive acute cutaneous graft versus host disease: A rare case report of survival* by Prajwal Pudasaini et al., https://doi.org/10.1002/ccr3.7545